# Optic Disc Vascularization in Glaucoma: Value of Spectral-Domain Optical Coherence Tomography Angiography

**DOI:** 10.1155/2016/6956717

**Published:** 2016-02-22

**Authors:** Pierre-Maxime Lévêque, Pierre Zéboulon, Emmanuelle Brasnu, Christophe Baudouin, Antoine Labbé

**Affiliations:** ^1^CHNO des Quinze-Vingts, DHU Sight Restore, INSERM-DHOS CIC, 28 rue de Charenton, 75012 Paris, France; ^2^Sorbonne Universités, UPMC, INSERM, CNRS, Institut de la Vision, 17 rue Moreau, 75012 Paris, France; ^3^Department of Ophthalmology, Ambroise Paré Hospital (AP-HP), University of Versailles Saint-Quentin-en-Yvelines, 55 Avenue de Paris, 78000 Versailles, France; ^4^Department of Ophthalmology III, Quinze-Vingts National Ophthalmology Hospital, 28 rue de Charenton, 75012 Paris, France

## Abstract

*Purpose*. To detect changes in optic nerve head (ONH) vascularization in glaucoma patients using spectral-domain OCT angiography (OCT-A).* Material and Method*. Fifty glaucoma patients and 30 normal subjects were evaluated with OCT-A (AngioVue®, Optovue). The total ONH vessel density and temporal disc vessel density were measured. Clinical data, visual field (VF) parameters, and spectral-domain OCT evaluation (RNFL: retinal nerve fiber layer thickness, GCC: ganglion cell complex thickness, and rim area) were recorded for glaucoma patients. Correlations among total and temporal ONH vessel density and structural and VF parameters were analyzed.* Results*. In the glaucoma group, total and temporal ONH vessel density were reduced by 24.7% (0.412 versus 0.547; *p* < 0.0001) and 22.88% (0.364 versus 0.472; *p* = 0.001), respectively, as compared with the control group. Univariate analysis showed significant correlation between rim area (mm^2^) and temporal ONH vessel density (*r* = 0.623; *p* < 0.0001) and total ONH vessel density (*r* = 0.609; *p* < 0.0001). Significant correlations were found between temporal and total ONH vessel density and RNFL, GCC, VF mean deviation, and visual field index.* Conclusion*. In glaucoma patients OCT-A might detect reduced ONH blood vessel density that is associated with structural and functional glaucomatous damage. OCT-A might become a useful tool for the evaluation of ONH microcirculation changes in glaucoma.

## 1. Introduction

Although elevated intraocular pressure (IOP) is the main risk factor for glaucoma, high numbers of patients also develop glaucoma with normal IOP levels [1]. Therefore, other risk factors and in particular vascular risk factors have been implicated in the pathogenesis of glaucoma [2]. These vascular factors include autoregulation of the blood flow (BF) in the optic nerve head (ONH), local vasospasm, arterial hypertension, and nocturnal hypotension [3]. Impaired microcirculation in the ONH may contribute to the initiation and progression of glaucomatous neuropathy. BF in the ONH is supplied by two arterial systems: the central retinal artery in superficial layers and the posterior ciliary artery in deeper layers (prelaminar and lamina cribrosa and retrolaminar structures) [4]. It has been proposed that the main pathologic changes in glaucoma are located in the deep ONH region, supplied by ciliary arteries [5].

Consequently, several techniques have been proposed to measure optic disc perfusion [6, 7]. Laser Doppler velocimetry has been used to identify the maximum BF velocity present in large retinal vessels [6]. Other methods such as scanning laser Doppler flowmetry [8] and laser speckle flowgraphy [9] were also developed to measure disc perfusion. Doppler optical coherence tomography (OCT) has been used to measure the total retinal blood flow around the ONH [10]. Using this imaging technique, BF in large vessels of the ONH can be quantified, but the study of the microvascular structure is impossible due to the low velocity of the BF in small vessels. The microsphere method, a method based on radioactive solid microspheres injected into the systemic circulation, has also provided evidence that the laser speckle flowgraphy is capable of assaying BF in the deep ONH region but only in experimental glaucoma models [11].

More recently, Jia et al. developed a new method to study in vivo the ONH microcirculation in glaucoma patients: the split spectrum amplitude-decorrelation angiography (SSADA) algorithm [12]. It provides high-quality three-dimensional (3D) angiography using ultra-high-speed swept-source OCT [13]. These authors found reduced disc perfusion in a group of patients with early glaucoma and established a link between ONH vessel density and visual field pattern standard deviation (PSD) using a custom swept-source OCT device [14]. The OCT angiography with SSADA may be more reliable than LSFG or LDF, offering better intravisit repeatability and intervisit reproducibility [14–16].

The objective of the present study was to detect changes in ONH vascularization by comparing normal subjects and glaucoma patients using a commercial spectral-domain OCT angiography (OCT-A) device.

## 2. Methods

### 2.1. Study Population

This cross-sectional observational study was conducted at the Quinze-Vingts National Ophthalmology Hospital, Paris, France, from January to September 2015 after approval of our Institutional Review Board. The study adhered to the tenets of the Declaration of Helsinki and informed consent was obtained from all subjects. Patients followed up at the Quinze-Vingts National Ophthalmology Hospital for glaucoma were consecutively included. Inclusion criteria were chronic glaucoma defined as glaucomatous optic disc neuropathy and characteristic visual field (VF) loss based on the criteria of the Ocular Hypertension Treatment Study [17]. The glaucomatous VF loss was defined as a glaucoma hemifield test graded “outside normal limits” and a cluster of three contiguous points at the 5% level on the pattern deviation plot, using the threshold test strategy with the 24-2 test pattern of the Humphrey Field Analyzer II [18]. Patients with diabetic retinopathy, other diseases that may cause visual field loss or optic disc abnormalities, and inability to clinically view or photograph the optic discs due to media opacity or poorly dilating pupil were excluded.

For all glaucoma patients, the following data were recorded: age, gender, best corrected visual acuity (BCVA) on a logarithmic scale, IOP (mmHg), central corneal thickness (CCT), type of glaucoma, and cup/disc ratio. IOP was measured using the Goldmann applanation tonometer. For each eye, IOP value represented the mean value of two IOP readings. CCT was measured with an A-scan ultrasound (A-Scan Pachymeter, Ultrasonic, Exton, PA, USA). The mean retinal nerve fiber layer (RNFL) thickness, mean ganglion cell complex (GCC) thickness, and disc rim area were also evaluated (Cirrus® spectral-domain OCT, Carl Zeiss Meditec Inc., Dublin, IE). VF tests were performed with a Humphrey Field Analyzer II (Carl Zeiss Meditec Inc.) set for 24-2 to collect mean deviation (MD), pattern standard deviation (PSD), and the visual field index (VFI). The Bascom Palmer (Hodapp-Anderson-Parrish) glaucoma staging system (GSS) based on VF results was selected to classify patients as having mild, moderate, and severe glaucoma [19].

Healthy age-matched controls were also included. Normal subjects had IOP < 21 mmHg with clinically normal ONH and no history of ocular or systemic disease. For control subjects, demographic data and IOP were recorded.

### 2.2. Optical Coherence Tomography

We used the commercially available spectral-domain OCT RT XR Avanti with the AngioVue software (Optovue, Inc., Fremont, CA, USA). It detects BF in an acquired volume using the SSADA [12]. The instrument used for OCT-A images is based on the AngioVue Imaging System (Optovue, Inc., Fremont, CA, USA) to obtain amplitude decorrelation angiography images. This instrument has an A-scan rate of 70,000 scans per second, using a light source centered on 840 nm and a bandwidth of 50 nm. Each OCT-A volume contains 304 × 304 A-scans with two consecutive B-scans captured at each fixed position before proceeding to the next sampling location. Split spectrum amplitude-decorrelation angiography was used to extract the OCT angiography information. Each OCT-A volume is acquired in three seconds and two orthogonal OCT-A volumes were acquired in order to perform motion correction to minimize motion artifacts arising from microsaccades and fixation changes.

The software analyzes the amplitude of variation of the OCT signal over time for every location acquired and calculates decorrelation. Static tissue yields low decorrelation values as the signal varies a little over time, and BF yields high decorrelation values because moving red blood cells cross the OCT beam and cause the signal amplitude to vary rapidly over time. A threshold decorrelation value is therefore used to discriminate BF from static tissue [20, 21]. The angiography information displayed was the average of the decorrelation values when viewed perpendicularly through the thickness evaluated. This maps all the detected vessels in the acquired volume. The AngioVue software (Optovue) can then calculate a quantitative variable: the total surface of the flowing vessel on the en face projection. The right eye from each normal subject was scanned. For glaucoma patients, we decided to analyze the most severe eye as determined by the MD 24-2.

### 2.3. Image Acquisition and Processing

First, each subject underwent one set of two scans on the macular region. The SSADA algorithm requires the user to define a threshold of detection of circulating vessels [13]. Jia et al. described a method to calculate the appropriate threshold. The central avascular zone was defined as a noise region and the mean decorrelation value of the whole avascular zone was calculated. A threshold one standard deviation above the mean avascular decorrelation value was defined as circulating vessels.

**Figure 1:**
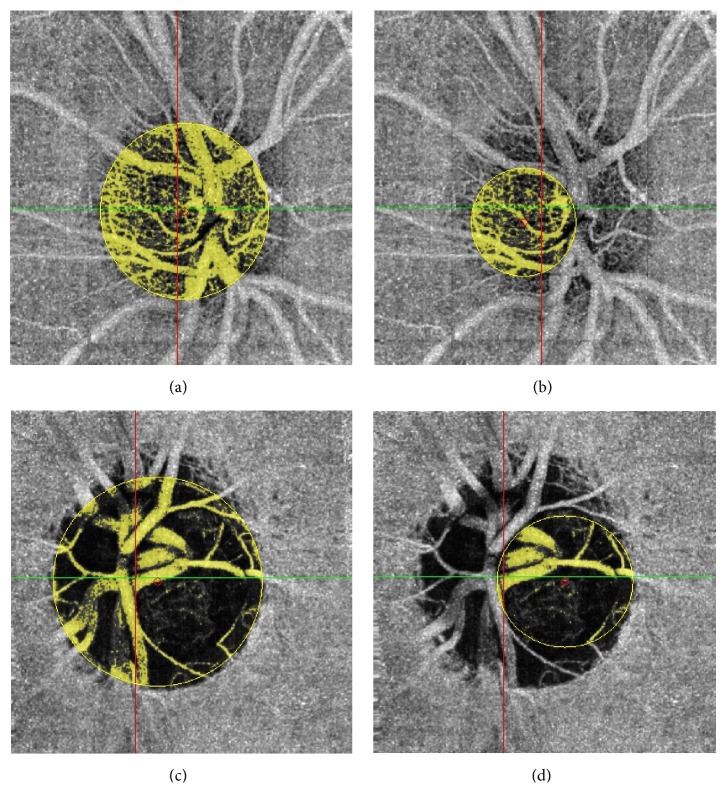
Total (a) and temporal (b) ONH acquisition in a normal patient. Total (c) and temporal (d) ONH acquisition in a glaucoma patient.

Then we performed one set of two 3 × 3 mm scans on the ONH. We used the neural canal opening, which is the termination of the retinal pigment epithelium/Bruch membrane complex, to define the ONH margin [22]. The volumes analyzed were defined by both a circular surface on the en face projection and the anteroposterior boundaries, thus creating a cylinder. A circle on the en face projection and anteroposterior limits defines each cylinder. We studied the total ONH flow area (mm^2^), representing the number of vessels, detected from all the depths of the disc, from the top of the ONH (inner surface) to the lamina cribrosa. Next, the total ONH vessel density (%) was calculated by dividing flow area by the optic disc surface (*πR* ^2^). The ONH temporal region (determined by the temporal disc between the temporal arteries and veins) was also studied to avoid BF due to major superior and inferior branches of retinal vessels in order to focus on the ONH laminar microvasculature (Figure 1). Then we measured the temporal disc flow area (mm^2^) and calculated the temporal disc vessel density by dividing the temporal disc flow area by the temporal disc surface (*πR* ^2^ temporal). The total and temporal optic disc vessel densities, obtained as the ratio of the optic disc flow area to the optic disc surface, were calculated to account for the intereye differences in total and temporal optic disc surface areas. Image quality was determined for all OCT-A scans. Poor quality images with a signal strength index (SSI) < 40 or image sets with remaining motion artifacts were excluded from the study analysis [23].

### 2.4. Reproducibility and Repeatability

Measurement repeatability of the disc flow area was evaluated from three sets of acquisitions from a subset of 6 normal subjects within a single visit. The intravisit coefficient of variation (CV) was calculated by comparing these three measurements. Intervisit reproducibility was obtained from three sets of scans performed on three separate visits from the same subset of normal subjects.

### 2.5. Statistical Analysis

Mann-Whitney tests were used to compare the average values of the measurements between normal and glaucomatous eyes. Linear regression analysis was used in the control group to investigate whether the measurement of ONH vessel density was affected by age, gender, and IOP. Univariate analysis with the Pearson correlation test and multivariate analysis with the ANCOVA test were performed to study the correlation between ONH vessel density and other variables: age, RNFL thickness, GCC, rim area, MD 24-2, and PSD 24-2.

## 3. Results

### 3.1. Demographic and Clinical Data

Fifty glaucoma patients and 30 normal subjects were included in this study. There was no difference between the two groups concerning age, gender, and ethnic origin. A family history of glaucoma was found in 52% of glaucoma patients and 10% of control subjects (*p* < 0.0001). IOP was 17.6 mmHg and 14.4 mmHg in the glaucoma and control group, respectively (*p* = 0.022). The demographic data are summarized in Table 1.

Regarding the type of glaucoma, 43 patients (86%) had open-angle glaucoma (OAG) and seven patients (14%) had angle-closure glaucoma (ACG). Twenty-three patients (46%) had mild glaucoma, eight (16%) had moderate glaucoma, and 19 (38%) had severe glaucoma. Mean RNFL thickness, GCC thickness, and rim area were 66.48 ± 12.6 *μ*m, 63.6 ± 9.07 *μ*m, and 0.786 ± 0.291 mm^2^ in the glaucoma group, respectively. The results of the glaucoma patients' clinical data are summarized in Table 2.

### 3.2. ONH Blood Flow Evaluation

The en face projection angiograms showed that normal discs had a denser microvascular network, especially in the temporal region, compared with glaucomatous discs (Figure 1). Total ONH vessel density was reduced by 24.7% (0.412 ± 0.117 versus 0.547 ± 0.077, *p* < 0.0001) in the glaucoma group as compared with the control group. Temporal disc vessel density was significantly reduced by 22.88% (0.364 ± 0.150 versus 0.472 ± 0.105, *p* = 0.001) in the glaucoma group as compared with the control group. Moreover, SSI and the decorrelation threshold were higher in the control group (72.3 versus 61.8, *p* < 0.0001, and 0.095 versus 0.085, *p* = 0.001, resp.). The intravisit CV was 6.17%. The intervisit CV was 6.48%. The results of the ONH BF evaluation are summarized in Table 3.

**Figure 2:**
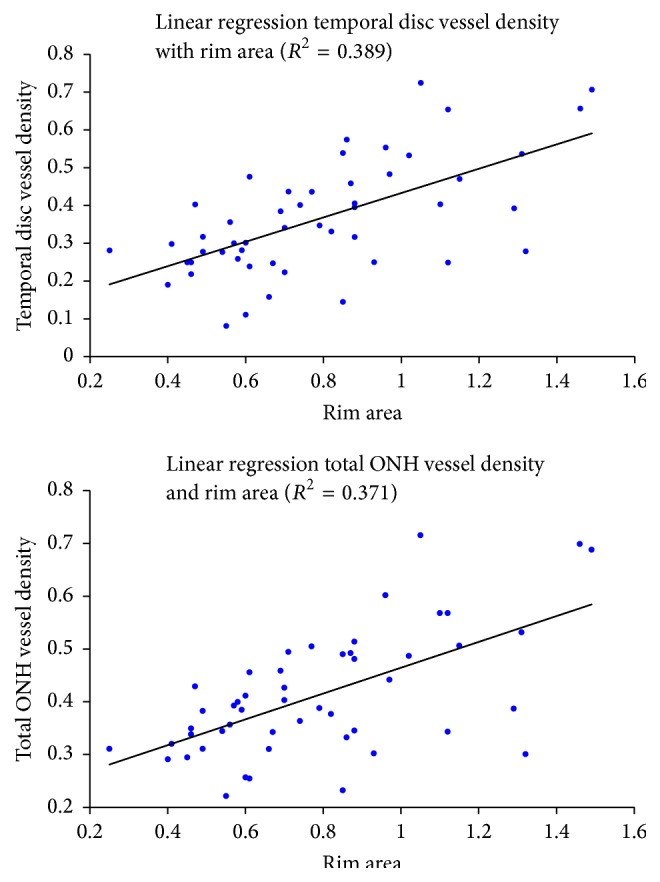
Linear regression analysis for temporal disc vessel density, total ONH vessel density, and rim area.

In the glaucoma group, univariate analysis showed that total ONH vessel density was significantly associated with structural variables including rim area (*r* = 0.609; *p* < 0.0001), RNFL thickness (*r* = 0.406; *p* < 0.003), and GCC thickness (*r* = 0.337; *p* = 0.017) (Figure 2). Total ONH vessel density was also associated with the functional variables VFI (*r* = 0.339; *p* = 0.017) and MD 24-2 (*r* = 0.301; *p* = 0.038). The results of these correlations are summarized in Table 4. In the multivariate analysis where total ONH vessel density was considered as a dependent variable, rim area was the dominant explanatory variable, accounting for 38.9% of the variance (*R* ^2^). In this multivariate model, RNFL thickness, GCC thickness, and VF parameters (MD 24-2 and VFI) were not statistically associated with ONH vascular density (Table 5).

Considering temporal disc vessel density, in univariate analysis, a correlation was observed with rim area (*r* = 0.624; *p* < 0.0001) (Figure 2), RNFL thickness (*r* = 0.448; *p* = 0.001), and GCC thickness (*r* = 0.395; *p* = 0.004). Temporal disc vessel density was also associated with VFI (*r* = 0.423; *p* = 0.002), MD 24-2 (*r* = 0.385; *p* = 0.007), but not with PSD 24-2 (Table 6). In a multivariate model studying temporal disc vessel density as a dependent variable, rim area was also the dominant explanatory variable, accounting for 39% of the variance (*R* ^2^). RNFL thickness, GCC thickness, and VF parameters (MD 24-2, VFI) were not associated with temporal disc vessel density (Table 7).

## 4. Discussion

In this study, we report the use of spectral-domain OCT angiography to measure ONH perfusion in glaucoma. We targeted two regions, the whole disc and a temporal disc area, to compare the two groups. We showed a significant difference in ONH vascular density between glaucoma and normal patients. The difference was similar for the total and temporal vascular area, with a decrease of ONH perfusion as compared with controls by 24.7% and 22.9%, respectively. We observed a significant correlation between ONH perfusion and structural and functional glaucoma damage. Moreover, the multivariate regression models confirmed the relation between disc rim area and ONH perfusion (total and temporal vessel density). These results emphasized the relation between ONH BF and structural loss in glaucoma.

In a previous similar study using a prototype of swept-source angio-OCT, Jia et al. studied ONH total BF with the SSADA. These authors found a correlation between the disc flow index and PSD, but they did not observe a correlation with structural variables such as the C/D ratio, rim area, or RNFL thickness [14]. Although altered microcirculation is reported to be associated with optic nerve head changes in glaucoma [24], these authors suggested that the flow index was independent information on glaucoma severity that is not explained by structural variables alone. The differences between our two studies can be explained by random variations due to the small sample size of the study reported by Jia et al. (24 normal subjects and 11 glaucoma patients). Moreover, most of their glaucoma patients had early-stage disease. In our study, 38% of the patients had severe glaucoma with advanced structural changes. The correlations we observed between ONH perfusion and structural parameters such as rim area, RNFL thickness, and GCC thickness could be explained by the more advanced glaucoma cases included in the present study. Interestingly, in these more advanced glaucoma patients, we also found a low but significant correlation with VF variables in univariate analysis, as did Jia et al. These results are also in accordance with the study by Liu et al. using OCT-A (RTVue-XR; Optovue, Inc.) that showed reduced peripapillary vessel density in glaucoma patients correlated with VF indexes [23]. In a recent study, Wang et al. comparing optic disc perfusion with OCT-A between 62 glaucoma patients and 20 control subjects found similar results with reduced disc vessel density in the glaucoma group correlated with structural parameters such as the GCC [25].

The SSADA algorithm requires the user to define a threshold of circulating vessel detection. Jia et al. described a method to calculate the appropriate threshold using the central avascular zone as a noise region and calculating the mean decorrelation value of the whole avascular zone. With their OCT-A prototype, they set the threshold two standard deviations above the mean avascular zone decorrelation. In the present study, using the commercially available spectral-domain OCT RT XR Avanti with the AngioVue software (Optovue, Inc. Fremont, CA, USA), we could not detect obvious circulating vessels using the same threshold as Jia et al. Therefore we set our threshold one standard deviation above the mean avascular decorrelation value. This difference might be explained by the difference in OCT-A hardware and software used in the two studies.

The results obtained with OCT-A confirmed the findings of several other techniques that reported ONH BF changes in glaucoma. Harris et al. measured ONH BF with different imaging technologies including color Doppler imaging, confocal scanning laser ophthalmoscopic angiography, laser blood flowmetry, or scanning laser Doppler flowmetry and demonstrated reduced ONH perfusion in glaucoma patients [6, 7]. Disc BF was also investigated by fluorescein angiography (FA) in several studies showing diffuse disc hypoperfusion and ONH fluorescein filling defects in glaucomatous patients [26]. Nevertheless, FA is not commonly used for monitoring glaucoma because of quantification problems and its potential side effects as an invasive technique [27]. Decreased ONH BF in glaucoma patients versus controls and increased viscosity in the papillary microvascular network were reported with the use of the laser Doppler velocimeter [28]. Using scanning laser Doppler flowmetry, Michelson et al. showed decreased peripapillary retinal BF and neuroretinal BF comparing glaucoma patients to control subjects [29]. Hafez et al. also found lower ONH BF in glaucoma patients and suggested a correlation between visual field defects and ONH perfusion [30]. Using laser speckle flowgraphy, other authors reported reduced BF in glaucoma patients and found a correlation between BF and MD and RNFL thickness [31]. Wang et al. confirmed a high correlation between the BF reduction measured by laser speckle flowgraphy and the microsphere method, providing evidence that this technique is capable of assaying BF for a critical deep ONH region [11].

The use of OCT-A to evaluate ONH BF may provide a new tool to investigate the role of the microvasculature in the pathophysiology of glaucoma and its changes with glaucoma progression. One of the longstanding questions is whether reduced BF is a preexisting pathologic change or a consequence of glaucomatous optic atrophy [32]. This is difficult to determine in part because a substantial portion of retinal ganglion cells/axons may have already become degenerated, even at an early stage of glaucoma [33]. Compromised blood supply and therefore reduced ocular BF due to increased IOP or vascular dysregulation has been implicated in the pathogenesis of the disease [7, 32]. In terms of laminar capillary volume flow, Burgoyne et al. hypothesized that, within the lamina, connective tissue IOP-related strain has both direct and indirect effects on axonal nutrition and that axonal ischemia can result from either IOP-induced occlusion of the laminar capillaries (direct effect) or decreased diffusion of nutrients (indirect effect), or both. In terms of only the retrolaminar effects on BF, the level of IOP-related strain within the peripapillary sclera may significantly affect volume flow through the scleral branches of the short posterior ciliary arteries. This hypothesis suggests that vascular mechanisms damaging the ONH are not necessarily IOP-independent and proposes a logic for understanding the complicated interaction between these three important factors (IOP, volume BF, and nutrient delivery) within the tissues of the ONH [34].

Our study has limitations that need to be considered when interpreting the results. Normal and glaucomatous eyes have been classified according to the visualization the ONH and no visual field was performed to control subjects. As OCT-A was used to evaluate ONH vascularization, we may have increased the chance of finding significant differences between groups. Similarly, by selecting the worst eye of glaucomatous patients we may have influenced the results. Nevertheless, this limitation has not influenced the analysis of correlation of OCT-A results and optic nerve parameters for glaucoma patients as this group was classified according to the results of VF.

The results of the present study suggest that spectral-domain OCT angiography might detect reduced ONH blood flow in glaucoma patients. These ONH blood flow changes were associated with structural and functional glaucomatous alterations. New studies are needed to evaluate the exact potential of OCT-A in glaucoma.

## Figures and Tables

**Table 1:** Qualitative and quantitative demographic data. IOP: intraocular pressure; F: female, M: male.

	Normal		Glaucoma		*p* value
Qualitative demographic data	(*n* = 30)	%	(*n* = 50)	%	

Gender					
F	17	56.7	23	46	NS
M	13	43.3	27	54	
Family history of glaucoma					
0	27	90	24	48	*p* < 0.0001
1	3	10	26	52	
Ethnic origin					
African	8	26.7	11	22	—
Caucasian	20	66.7	33	66	
Asian	0	0	2	4	
Hispanic	0	0	2	4	
Maghrebi	2	6.7	2	4	

Quantitative demographic data	Mean	SD	Mean	SD	

Age (years)	51.1	18.1	58.4	15.9	NS
IOP (mmHg)	14.4	2.59	17.6	6.41	0.022

**Table 2:** Quantitative and qualitative clinical data. OAG: open-angle glaucoma; ACG: angle-closure glaucoma. Visual field and OCT spectral-domain testing. MD: mean deviation; PSD: pattern standard deviation; VFI: visual field index; RNFL: retinal nerve fiber layers; GCC: ganglion cell complex.

	Glaucoma	
Qualitative clinical data	(*n* = 50)	%

Type		
OAG	43	86
ACG	7	14
Stage		
Mild	23	46
Moderate	8	16
Severe	19	38

Quantitative clinical data	Mean	SD

log AV	0.142	0.239
Corneal central thickness (*µ*m)	525.8	46
Cup/disc ratio	0.821	0.153
MD 24-2 (dB)	−10.5	8.91
PSD 24-2 (dB)	6.81	4.7
VFI	70.87	27.6
RNFL thickness (*µ*m)	66.48	12.6
GCC thickness (*µ*m)	63.6	9.07
Rim area (mm^2^)	0.786	0.29

**Table 3:** OCT angiography measurements. SSI: strength signal index.

OCT angiography measurements	Normal		Glaucoma		*p* value
	Mean	SD	Mean	SD	
Threshold	0.095	0.012	0.085	0.012	0.001
SSI	72.3	8.41	61.8	7	<0.0001
Total ONH vessel density	0.547	0.154	0.412	0.117	<0.0001
Temporal disc vessel density	0.472	0.105	0.364	0.15	0.0001

Glaucoma stage	Total ONH vessel density		Temporal disc vessel density		
	Mean	SD	Mean	SD	

Mild	0.441	0.145	0.404	0.178	
Moderate	0.439	0.067	0.414	0.110	
Severe	0.365	0.076	0.293	0.096	

**Table 4:** Pearson correlation coefficient matrix for visual field, structural variables, and ONH total vessel density in subjects with glaucoma. MD: mean deviation; PSD: pattern standard deviation; VFI: visual field index; RNFL: retinal nerve fiber layers; GCC: ganglion cell complex; total ONH density: total ONH vessel density; SSI: strength signal index. Statistically significant correlations with total ONH vessel density are in bold.

Variable		Total ONH density	SSI	VFI	PSD 24-2	MD 24-2	GCC	Rim area	RNFL	Age
Total ONH density	*r*	**1**	** **	** **		** **	** **	** **	** **	
	*p*	**0**	** **	** **		** **	** **	** **	** **	
SSI	*r*	**0.318**	1							
	*p*	**0.026**	0							
VFI	*r*	**0.339**	0.248	1						
	*p*	**0.017**	0.089	0						
PSD 24-2	*r*	−0.192	−0.090	−0.595	1					
	*p*	0.185	0.544	<0.0001	0					
MD 24-2	*r*	**0.301**	0.202	0.986	−0.685	1				
	*p*	**0.038**	0.173	<0.0001	<0.0001	0				
GCC thickness	*r*	**0.337**	0.230	0.580	−0.472	0.532	1			
	*p*	**0.017**	0.111	<0.0001	0.001	0.000	0			
Rim area	*r*	**0.609**	0.187	0.622	−0.581	0.628	0.598	1		
	*p*	**<0.0001**	0.198	<0.0001	<0.0001	<0.0001	<0.0001	0		
RNFL thickness	*r*	**0.406**	0.201	0.649	−0.533	0.664	0.789	0.661	1	
	*p*	**0.003**	0.166	<0.0001	<0.0001	<0.0001	<0.0001	<0.0001	0	
Age	*r*	0.166	−0.308	0.268	−0.285	0.350	0.047	0.267	0.244	1
	*p*	0.248	0.031	0.063	0.047	0.015	0.746	0.061	0.088	0

**Table 5:** Multivariate model for total ONH vessel density *R* ^2^ = 0.387.

Variable	Slope	*p* value
MD 24-2	−0.002	0.870
VFI	−0.001	0.955
GCC thickness	−0.002	0.484
Rim area	**0.269**	**0.000**
RNFL thickness	0.002	0.445

MD: mean deviation; VFI: visual field index; RNFL: retinal nerve fiber layers; GCC: ganglion cell complex. Statistically significant correlations with total ONH vessel density are in bold.

**Table 6:** Pearson correlation coefficient matrix for visual field, structural variables, and temporal disc vessel density in subjects with glaucoma. MD: mean deviation; PSD: pattern standard deviation; VFI: visual field index; RNFL: retinal nerve fiber layers; GCC: ganglion cell complex; SSI: strength signal index. Statistically significant correlations with temporal disc vessel density are in bold.

Variable		Temporal disc density	SSI	VFI	PSD 24-2	MD 24-2	GCC	Rim area	RNFL	Age
Temporal disc density	*r*	**1**		** **		** **	** **	** **	** **	
	*p*	**0**								
SSI	*r*	0.235	1							
	*p*	0.105	0							
VFI	*r*	**0.423**	0.248	1						
	*p*	**0.002**	0.089	0						
PSD 24-2	*r*	−0.171	−0.090	−0.595	1					
	*p*	0.241	0.544	<0.0001	0					
MD 24-2	*r*	**0.385**	0.202	0.986	−0.685	1				
	*p*	**0.007**	0.173	<0.0001	<0.0001	0				
GCC thickness	*r*	**0.396**	0.230	0.580	−0.472	0.532	1			
	*p*	**0.004**	0.111	<0.0001	0.001	0.000	0			
Rim area	*r*	**0.624**	0.187	0.622	−0.581	0.628	0.598	1		
	*p*	**<0.0001**	0.198	<0.0001	<0.0001	<0.0001	<0.0001	0		
RNFL thickness	*r*	**0.448**	0.201	0.649	−0.533	0.664	0.789	0.661	1	
	*p*	**0.001**	0.198	<0.0001	<0.0001	<0.0001	<0.0001	<0.0001	0	
Age	*r*	0.185	−0.308	0.268	−0.285	0.350	0.047	0.267	0.244	1
	*p*	0.199	0.031	0.063	0.047	0.015	0.746	0.061	0.088	0

**Table 7:** Multivariate model for temporal disc vessel density *R* ^2^ = 0.390.

Variable	Slope	*p* value
MD 24-2	0.005	0.717
VFI	−0.002	0.680
GCC thickness	−0.001	0.690
Rim area	**0.311**	**0.001**
RNFL thickness	0.001	0.648

MD: mean deviation; VFI: visual field index; RNFL: retinal nerve fiber layers; GCC: ganglion cell complex. Statistically significant correlations with temporal disc vessel density are in bold.
